# The Trinity of Skin: Skin Homeostasis as a Neuro–Endocrine–Immune Organ

**DOI:** 10.3390/life12050725

**Published:** 2022-05-12

**Authors:** Rong Jin, Lan Luo, Jie Zheng

**Affiliations:** 1 Department of Dermatology, Rui Jin Hospital, Shanghai Jiao Tong University School of Medicine, Shanghai 200025, China; avisj@foxmail.com; 2 Department of Plastic and Reconstructive Surgery, Shanghai Ninth People’s Hospital, Shanghai Jiao Tong University School of Medicine, Shanghai 200025, China; roland_rainbow@outlook.com

**Keywords:** skin, neuro–endocrine–immune, homeostasis, rosacea

## Abstract

For a long time, skin was thought to be no more than the barrier of our body. However, in the last few decades, studies into the idea of skin as an independent functional organ have gradually deepened our understanding of skin and its functions. In this review, we gathered evidence that presented skin as a “trinity” of neuro–endocrine–immune function. From a neuro perspective, skin communicates through nerves and receptors, releasing neurotrophins and neuropeptides; from an endocrine perspective, skin is able to receive and secrete most hormones and has the cutaneous equivalent of the hypothalamic-pituitary-adrenal (HPA) axis; from an immune perspective, skin is protected not only by its physical barrier, but also immune cells and molecules, which can also cause inflammation. Together as an organ, skin works bidirectionally by operating peripheral neuro–endocrine–immune function and being regulated by the central nervous system, endocrine system and immune system at the same time, maintaining homeostasis. Additionally, to further explain the “trinity” of cutaneous neuro–endocrine–immune function and how it works in disease pathophysiology, a disease model of rosacea is presented.

## 1. Introduction

The skin is the largest organ of the human body, protecting internal homeostasis from the external environment. However, skin is not only a simple barrier, but also involved in maintaining internal homeostasis through bidirectional communications between the central nervous, endocrine and immune systems. As far back as 1998, the idea of a neuro–immune–cutaneous–endocrine network was developed and initialized as “NICE” [1], although the endocrine aspect was not elucidated in these early articles. Shifting our focus to the present day, emerging evidence has gradually verified that the skin shares and provides the same bioactive molecules as the body, especially the evidence of cutaneous production and action of neuropeptides, hormones and cytokines, which will be discussed in this review. This suggests the existence of cross-talk between the skin and the system, and gives the skin a new identity of a neuro–endocrine–immune organ. In this review, we gathered evidence that indicated skin functioning as a nervous, endocrine or immune organ, connected with the central nervous, endocrine and immune systems. Furthermore, a disease model of rosacea was used to further explain skin as a “trinity” of a neuro–endocrine–immune organ and its participation in pathogenesis.

## 2. Neuro Function of Skin

The term “neurogenic inflammation” suggests the critical role of the cutaneous nervous system in immune response and homeostasis. Chronic inflammatory skin diseases such as atopic dermatitis (AD) and psoriasis can be aggravated by stress [2,3,4,5,6], which is a good example of neurogenic inflammation. In this chapter, the basic cutaneous nervous system anatomy and communication between the cutaneous nervous and immune system will be explained. On the other hand, the relationship between the cutaneous nervous and endocrine system will be reviewed in the next chapter.

### 2.1. Anatomic Foundation of Cutaneous Nervous System

Skin is derived from ectoderm, like the nervous system, which makes it easier to understand the diverse nervous function of skin. Skin is innervated with mostly sensory nerves, classified into A-β, A-δ and C fibers according to their diameter, myelinization, and velocity of conduction [7]. A-β fibers are highly myelinated, rapid conducting fibers and innervating specialized mechanosensory end organs that include Meissner’s corpuscles, Pacinian corpuscles, Merkel cells, and Ruffini corpuscles [8,9]. A-δ fibers are less myelinated fibers with slower conduction velocity that give sensation to mechanical, heat nociception and non-noxious cold thermal stimuli. C fibers are unmyelinated fibers with the lowest conducting speed, precept thermal and chemical and mechanical stimuli [10,11,12]. Along these fibers lies immune cells such as mast cells [13,14], dendritic cells [15,16], macrophages [17], innate lymphoid cells [18] and γδT cells [19], forming neuroimmune cell units (NICUs) that orchestrate skin homeostasis [20,21].

### 2.2. Neuroimmune Interactions of Skin

The cutaneous nervous system and immune system have a responsibility in common: sensing. Whether recognizing pathogens through immune cells or precepting noxious stimuli via sensory nerves, these two “sensing” systems of the skin work synergistically against environmental challenges.

Upon sensing stimuli, especially noxious ones, the cutaneous nervous system tends to communicate with the immune system via neurotrophins (NTs) and neuropeptides (NPs), causing subsequent cascading effects known as “neuroimmune interactions” [22,23,24,25]. NTs belong to a family of growth factors that control the development, maintenance, and apoptosis of neurons and regulate skin homeostasis; for example, the stimulation of mast cell degranulation and cytokine release [26]. NPs, such as substance P (SP), calcitonin gene-related protein (CGRP) and hundreds of other types, are secreted by cutaneous nerves [27]. SP induces mast cell degranulation and the release of histamine and vascular endothelial growth factor (VEGF), subsequently causing proinflammatory effects, hypervascularization and infiltration of inflammatory cells [28,29]. CGRP is involved in vasodilation and neurogenic inflammation [30].

However, neuroimmune reaction of the skin is bidirectional. The cutaneous nervous system also takes orders from the immune system through cytokines. Immune cells sense pathogenic events though a set of receptors, recognizing pathogen-associated molecular patterns (PAMPs) such as LPS and CpG, and damage-associated molecular patterns (DAMPs); for instance, HMGB1, S100 proteins and heat-shock proteins [31,32,33]. Such pattern-recognition receptors (PRRs) such as Toll-like receptors (TLRs) and IL-1R, after binding with PAMPs and DAMPs, lead to inflammatory and immune responses through signaling to nuclear factor kB (NF-kB) [34], inducing the expression of proinflammatory cytokines such as IL-1, -6, -31, IFN-I and TNF-α [35,36]. With cytokine serving as ligands and activators of sensory nerves [37], downstream neuro effects take place. For example, IL-6 induces the expression of nerve growth factor (NGF) and NT-3, 4, and 5 [38,39], while IL-31 exerts its pruritic effects [40].

### 2.3. Skin-CNS Connection

The cutaneous nervous system, as part of the peripheral nervous system, sends and receives messages from the central nervous system (CNS), which can be elucidated using the model of pruritus. Itch receptors on neuropeptide-containing free nerve endings can be directly set off by histamine and other pruritogens, or indirectly by cytokine-induced histamine release [41]. Once an action potential is set off, it travels through the dorsal root ganglia onto the spinal cord, eventually to the somatosensory cortex in the brain. Conversely, CNS also participate in the modulation and inhibition of peripheral pruritis via periaqueductal grey matter (PAG) of the mid-brain [42,43]. Additionally, psychological stress can aggravate pruritus [44,45], which is another solid evidence of skin–CNS connection.

## 3. Endocrine Function of Skin

### 3.1. Skin as Endocrine End Organ

Skin is the target of several hormones and expresses a number of endocrine receptors. For example, glucocorticoids (GCs) and mineralocorticoids (MCs) interfere with the epidermal development and homeostasis through GC receptor (GR/NR3C1) and mineralocorticoid receptor (MR/NR3C2), both of which are members of the nuclear receptor (NR) subclass NR3C and are present in all skin compartments [46]. Like GCs and MCs, thyroid hormones (THs) also participate in epidermal development and homeostasis via nuclear thyroid hormone receptors (TRs) TRα and TRβ, expressed in epidermal and dermal cells [47]. Androgen receptors in sebaceous glands and hair follicles regulate sebum secretion and hair growth [48]; insulin receptor and insulin-like growth factor 1 (IGF-1) receptor in keratinocytes (KCs) modify cutaneous development and metabolism [49]. In conclusion, various kinds of hormones bring their biological effects into skin through binding with cutaneous endocrine receptors. Therefore, when the systemic endocrine function is compromised, cutaneous homeostasis will also be influenced. There are abundant supporting clinical findings such as the correlation between acanthosis nigricans, acrochordon and metabolic syndrome in patients with lichen planus [50]; the connection between alopecia areata, vitiligo and autoimmune thyroid disease [51]; and the relationship between acne, hypertrichosis and insulin resistance [52].

### 3.2. Skin as Endocrine Initiating Organ

Hormones are synthesized in skin mainly through two ways: activation of circulating hormone precursors and de novo synthesis. Examples of the former way include the activation of cortisol and corticosterone through local 11β-hydroxysteroid dehydrogenase (11β-HSD) [53] and the intracellular T4 conversion into T3 via iodothyronine deiodinase enzymes D1 and D2 [47]. Both GCs and THs are essential for skin homeostasis, GCs downregulate inflammation [54] while THs enhance skin susceptibility to inflammation [55]. Another well-known example of hormone activation is the conversion of dehydroepiandrosterone (DHEA) to androstenedione, then to testosterone through isotypes of 17β-hydroxysteroid dehydrogenase (17β-HSD) in skin. Further conversion of testosterone into its most potent form, 5α-dihydrotestosterone (5α-DHT), is completed by 5α-reductase in skin [56].

Other than in traditional endocrine organs, de novo synthesis of hormones also takes place in skin. The most studied example is the equivalent of hypothalamic-pituitary-adrenal (HPA) axis in skin. The traditional adaptive responses to systemic stress are regulated by the HPA axis. Activation of the traditional HPA axis begins with the pituitary production of the corticotropin-releasing hormone (CRH) following stimulation by corticotrophin-releasing factor (CRF) secreted from the hypothalamus. Then, CRH receptor type 1 in the anterior pituitary is activated and induces the cleavage of proopiomelanocortin (POMC) into the adrenocorticotropic hormone (ACTH), α-melanocyte-stimulating hormone (α-MSH) and β-endorphin (β-END) [57]. ACTH stimulates the adrenal cortex to secret GCs, which responds to stressors and suppresses the HPA axis through negative feedback [57].

When stressors come to the skin, KCs produce hormonal products, similar to that produced in systemic stressful events such as CRH, POMC, β-END, ACTH and α-MSH [58]. Moreover, enzymes of corticosteroid synthesis such as CYP11A1, 3β-HSD, CPY17A1, CYP21A2 and CYP11B1 are expressed in KCs and thus produce corticosterone and cortisol [59,60], which further proves the existence of the skin HPA axis. In an IMQ-treated mouse model whose KC-derived CYP11B1 was knocked out, local homeostasis was impaired and psoriasiform inflammation was exacerbated. Furthermore, even non-IMQ-treated mice presented psoriasiform inflammation after CYP11B1 knock out, showing the homeostasis stabilizing effect of KC-derived GC and the importance of the whole skin equivalent of the HPA axis [54].

Apart from the well-known GCs, vitamin D and its analogs are known as secosteroids, which is synthesized from the skin. In KCs of the basal layer of epidermis, 7-dehydrocholesterol (7-DHC) is converted to vitamin D3 under UVB light, then released into system to further undergo biological activation in the hepatocytes and kidneys [61]. Numerous skin functions are regulated by vitamin D and its receptor, including coregulation in epidermal proliferation and differentiation [62], regulation of the hair follicle cycle [63], promotion of innate immunity [64], and suppression of tumor formation and inflammation [65]. Vitamin D disturbance is often seen in skin diseases, such as low vitamin D status in psoriasis [66] and chronic urticaria patients [67], and elevated vitamin D level in rosacea patients [68].

## 4. Immune Function of Skin

### 4.1. Barrier and Immune Cells Undernease

Unlike intestinal and pulmonary mucosa, which only have one single layer of epithelial barrier, the skin barrier acts as “bricks and mortar”—keratinocytes (KCs) as “bricks” and intercellular matrix as “mortar”. Such a firm and tight structure forms the physical barrier and protects internal organs from external hazards [69]. Other than being components of a physical barrier, KCs themselves have innate immune features and the ability to induce adapted immune response. As the first sensors and immune sentinels of pathogen invasion, KCs can recognize nonspecific external stimuli such as microbial ligands, UV rays and chemicals via receptors such as TLRs, TNF-α receptor 1 (TNFR1) and IL-1R [70]. Responding to stimuli, KCs produce various cytokines, chemokines, growth factors and antimicrobial peptides (AMPs), leading to either direct neutralization of the pathogen or indirect activation of other specific immune responses [71].

Underneath the barrier lies various immune cells, such as Langerhans cells (LCs), dendritic cells (DCs), mast cells (MCs), B and T lymphocytes, together with skin cells that constitute skin-associated lymphoid tissue (SALT) [72]. Under a steady state, these immune cells surveillance skin homeostasis and help maintain a balanced metabolism and barrier integrity [73,74,75]. When homeostasis is broken, SALT elicits it effect by recognizing the pathogen, modulating the cascade of the local immune responses and participating in the pathophysiology of autoimmune and hypersensitivity disorders [76]. The term inducible SALT (iSALT) was further created to describe the skin immune cell complex under the elicitation phase, which does not present under steady homeostasis [77]. The iSALT provides an antigen presentation site in the skin, which is critical for elicitation of adaptive immunity such as T cell activation [16].

### 4.2. Systemic Association with Cutaneous Immue System

Skin-derived immune cells not only modulate local immune response but are also involved in systemic inflammation through cytokine release and cell migration. For example, KCs can produce a plethora of cytokines such as IL-1, -6, -7, -8, -10, -12, -15, -18, -20, and TNF-α, causing proinflammatory and anti-inflammatory effects [78]. Immune cells including LCs [79], DCs [80], MCs [81], and T lymphocytes [82] are found able to migrate from skin to draining lymph nodes, further affecting systemic immune response.

γδT cells, which are the key pathogenic cells of psoriasis, have been suggested to be a potential candidate contributing to the development of psoriatic cardiovascular disease [83]. In a psoriasis mouse model, γδT cells in the skin migrate to the draining lymph nodes and re-appear when the skin is exposed to imiquimod (IMQ) again. The migration relies on the CCR6-CCL20 pathway, meanwhile CCL20 is found upregulated in hypertension damaged vessel walls and the plaque of atherosclerosis [84]. This suggests the migration of γδT cells may contribute to psoriatic cardiovascular disease, pointing out the potential reason of “psoriatic march” [83]. Other than γδT cells and CCL20, a common concept of psoriatic march believes that the release of excessive proinflammatory cytokines such as TNF-α and IL-1 in psoriasis causes chronic low-grade systemic inflammation, leading to insulin resistance, visceral adiposity, hypertension and dyslipidemia, and, finally, the development of type 2 diabetes and cardiovascular disease [85].

Besides psoriatic march, another much-discussed cutaneous inflammation that can cause systemic impact is the case of “atopic march”. When the skin barrier is impaired due to inflammation in AD, allergens are exposed to cutaneous immune cells, which induces sensitization and promotes the development of specific T and B cell responses, causing subsequent allergic disease [86]. Cutaneous DCs and other immune cells migrate to draining lymph nodes and induce differentiation of naive T cells into allergen-specific TH2 cells [87]. These TH2 cells can exert immune effects, systemically affecting the lungs, esophagus, and gastrointestinal tract, causing systemic allergic disease [86].

In contrast to the two inflammatory skin marches mentioned above, systemic immune cells can also act on skin through chemotaxis and homing, showing bidirectional characteristics between the systemic and cutaneous immune system. In steady homeostasis, T cells patrol peripheral tissues such as skin to facilitate swift immune responses against pathogens. Once homeostasis is imbalanced, chemokines are expressed during immune response to combine with chemokine receptors on T cells, thus chemotaxis and homing take place [88]. Such a process participates in the pathogenesis of AD [89], psoriasis [90], alopecia areata [91], vitiligo [92], rosacea [93] and cutaneous T cell lymphoma (CTCL) [94].

## 5. Rosacea as a Disease Model of the Trinity of Skin

To further explain skin as a neuro–endocrine–immune organ, a clinical related disease model is required. One well-studied example, psoriasis, is driven by innate immune cells, adaptive immune cells, keratinocytes and their production of cytokines. Once stressed environmentally or psychologically, activated HPA axis and secreted neuropeptides may exacerbate the progression of psoriasis, where neuro–endocrine–immune functions meet together. Since the neuro–endocrine–immune connection of psoriasis is so well-discussed elsewhere [95,96,97], the details will not be described in this review. Instead, we will focus on another inflammatory dermatosis: rosacea.

Although the exact pathogenesis of rosacea still remains unclear, rosacea is a common disorder with a pathogenesis that involves immune dysfunction, neurovascular dysregulation and stress hormones that can be a good illustration of the trinity of the skin. Like many other inflammatory skin diseases, rosacea starts with the sensing of external physical, chemical and biological stimulations by the cutaneous nervous and immune system. Sensory neuron density is increased in rosacea [98], while immune cells such as mast cells have been found in increased numbers in rosacea-affected skin [99].

Physical stimuli such as temperature changes and chemical stimuli such as spices can activate sensory nerves through the transient receptor potential (TRP) family of cation channels [100]. Once TRP ion channels are activated, vasoactive neuropeptides such as SP, CGRP and vasoactive intestinal peptide (VIP) are released, resulting in enhanced skin blood flow and telangiectasia [23,98]. SP can further induce mast cell degranulation, causing increased levels of proinflammatory cytokines such as IL1, IL2, IL6 and TNF-a, chemokines such as CCL2, CCL5, CXCL8, CXCL9 and CXCL10 [101], leading to neurogenic inflammation in rosacea.

On the other hand, biological stimuli, namely PAMPs produced by staphylococcus, demodex or other pathogens, and DAMPs caused by damages, are sensed by TLRs, inducing conserved anti-pathogen signaling pathways, including the production of antimicrobial peptides (AMPs) such as cathelicidin and proinflammatory cytokines and chemokines [102]. Cathelicidin is further cleaved into LL-37, its active peptide form, by kallikrein 5 (KLK5), causing leukocyte chemotaxis, activation of NF-kB and promotion of angiogenesis [103,104], resulting in morphologic changes in rosacea, such as telangiectasia, facial erythema, papules and pustules.

CRH also plays a critical role in rosacea pathogenesis. When skin is stressed by external physical, chemical and biological stimuli, or by systemic psychological and physical stress, CRH is either released from the pituitary or expressed in the skin [105]. Even a physiological dose of UVB radiation can increase CRH synthesis significantly in KCs, sebocytes and fibroblasts [106]. CRH acts as the central coordinator for neuro–endocrine–immune responses, causes degranulation of mast cells and notable increases in vascular permeability, regulates IL18 secretion in KCs and IL6, IL8 production in sebocytes, which mediate MAP kinase (MAPK) and NF-kB, and may lead to inflammation and facial erythema [107]. In addition, CRH might also increase the expression of TLRs [108] and activate cannabinoid and vanilloid pathways [109].

**Figure 1:**
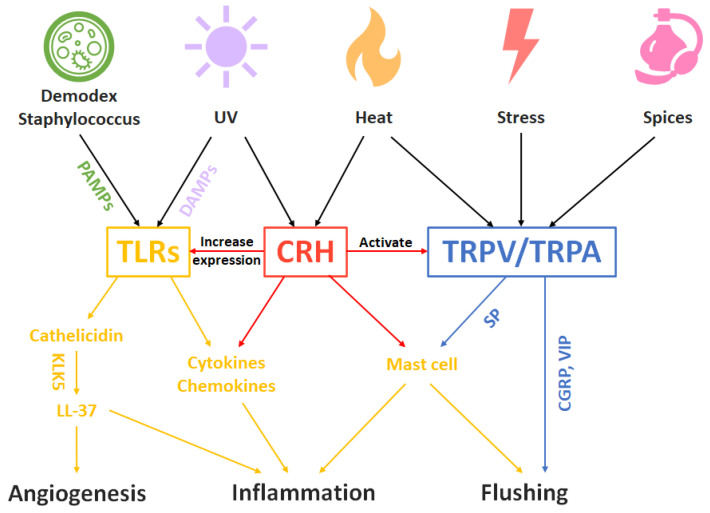
Rosacea disease model of cutaneous neuro–endocrine–immune system. The TLRs represent the sentinel of cutaneous immune system. The CRH represents the action of cutaneous endocrine system. The TRPV/TRPA represents the sentinel of cutaneous nervous system. The parts belongs to cutaneous nervous, endocrine and immune system are colored in blue, red and yellow, respectively. The combination of disruption of the cutaneous neuro–endocrine–immune system results in the final clinical manifestation of rosacea. Abbreviations: CGRP: calcitonin gene-related protein; CRH: corticotropin releasing hormone; DAMPs: damage-associated molecular patterns; KLK5: kallikrein 5; SP: substance P; TLRs: Toll-like receptors; TRPV/TRPA: transient receptor potential vanilloid/transient receptor potential ankyrin; UV: ultraviolet; VIP: vasoactive intestinal peptide.

To summarize, in the rosacea model of the neuro–endocrine–immune trinity of skin, the pathogenesis is started with extrinsic or intrinsic stress and stimuli, undergoes the regulation and participation of cutaneous and systemic nervous, endocrine and immune system, and results in inflammation and morphologic changes (Figure 1).

## 6. Discussion

**Figure 2:**
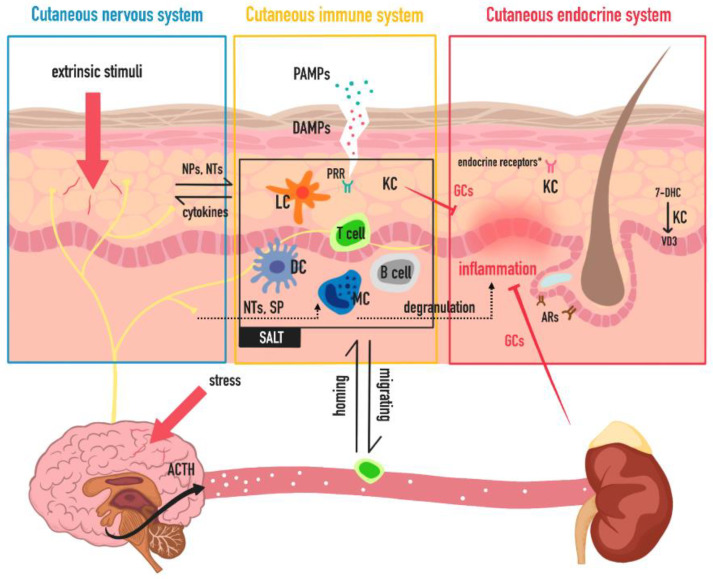
Skin as a “trinity” of a neuro–endocrine–immune organ. In this illustration, skin is divided into cutaneous nervous, endocrine and immune system. Cutaneous nervous system senses extrinsic stimuli via sensory nerves and receptors, communicates with CNS and provides NPs, NTs which can stimulate immune cells [23]. Cutaneous immune system is composed of innate immune cells, adaptive immune cells and KCs. It senses PAMPs and DAMPs through PRRs on KCs and innate immune cells [110], participates in inflammation and communicate with systemic immune system through cytokines, immune cell migrating and homing. Cutaneous endocrine system, especially the de novo hormone synthesis “plant”, KCs, is able to synthesize GCs [59] and vitamin D [61], regulate local and systemic homeostasis. Endocrine receptors*: including glucocorticoid receptors, mineralocorticoid receptor, thyroid hormone receptors, insulin receptors and insulin-like growth factor 1 (IGF-1) receptors, etc. Abbreviations: ACTH: adrenocorticotropic hormone; ARs: androgen receptors; CNS: central nervous system; DAMPs: damage-associated molecular patterns; DC: dendritic cell; GCs: glucocorticoids; KC: keratinocyte; LC: Langerhans cell; MC: mast cell; NPs: neuropeptides; NTs: neurotrophins; PAMPs: pathogen-associated molecular patterns; PRR: pattern-recognition receptors; SALT: skin-associated lymphoid tissue; SP: substance P, VD3: vitamin D3; 7-DHC: 7-dehydrocholesterol.

To conclude this review, a brief outline of the skin as a neuro–endocrine–immune organ and its function involved in maintaining homeostasis is made below (Figure 2).

It might be noted that the word “neuro–endocrine–immune” is ended with “immune” in this review, different to most articles where “neuro-immuno-endocrine” is used instead [37,111]. This is because, most of the time, the cutaneous immune system plays the final role in pathogenesis through inflammation, and also has the ability to sense pathogens and initiate a defensive reaction itself.

There is plenty of evidence supporting the concept that skin is a neuro–endocrine–immune organ, including but not limited to the neuroanatomy of skin, the production of neurotrophins and neuropeptides, the cutaneous equivalent of HPA axis, and the SALT with its production of cytokines and chemokines. All three aspects of functions together as a trinity keeps the skin in a steady state. Any disruption to the skin could result in a fall in local homeostasis, further influencing systemic homeostasis such as psoriatic march and atopic march. Hence, maintaining the integrity of the skin barrier and keeping it under homeostasis is critical. Further research on the neuro–endocrine–immune function of the skin might provide new perspectives on the pathogenesis of skin diseases in view of a bigger picture and give a rise to new therapeutic options.

## Data Availability

Data sharing not applicable.

## References

[1] O’Sullivan R.L., Lipper G., Lerner E.A. (1998). The neuro-immuno-cutaneous-endocrine network: Relationship of mind and skin. Arch. Dermatol..

[2] Lin T.K., Zhong L., Santiago J.L. (2017). Association between Stress and the HPA Axis in the Atopic Dermatitis. Int. J. Mol. Sci..

[3] Mochizuki H., Lavery M.J., Nattkemper L.A., Albornoz C., Valdes Rodriguez R., Stull C., Weaver L., Hamsher J., Sanders K.M., Chan Y.H. (2019). Impact of acute stress on itch sensation and scratching behaviour in patients with atopic dermatitis and healthy controls. Br. J. Dermatol..

[4] Yang H., Li X., Zhang L., Xue F., Zheng J. (2019). Immunomodulatory effects of sleep deprivation at different timing of psoriasiform process on skin inflammation. Biochem. Biophys. Res. Commun..

[5] Yang H., Zheng J. (2020). Influence of stress on the development of psoriasis. Clin. Exp. Dermatol..

[6] Bozo R., Danis J., Flink L.B., Vidacs D.L., Kemeny L., Bata-Csorgo Z. (2021). Stress-Related Regulation Is Abnormal in the Psoriatic Uninvolved Skin. Life.

[7] Glatte P., Buchmann S.J., Hijazi M.M., Illigens B.M., Siepmann T. (2019). Architecture of the Cutaneous Autonomic Nervous System. Front. Neurol..

[8] Fleming M.S., Luo W. (2013). The anatomy, function, and development of mammalian Abeta low-threshold mechanoreceptors. Front. Biol..

[9] Cobo R., Garcia-Piqueras J., Cobo J., Vega J.A. (2021). The Human Cutaneous Sensory Corpuscles: An Update. J. Clin. Med..

[10] Lawson S.N. (2002). Phenotype and Function of Somatic Primary Afferent Nociceptive Neurones with C-, Adelta- or Aalpha/beta-Fibres. Exp. Physiol..

[11] Djouhri L. (2016). Adelta-fiber low threshold mechanoreceptors innervating mammalian hairy skin: A review of their receptive, electrophysiological and cytochemical properties in relation to Adelta-fiber high threshold mechanoreceptors. Neurosci. Biobehav. Rev..

[12] Schmelz M., Schmidt R., Weidner C., Hilliges M., Torebjork H.E., Handwerker H.O. (2003). Chemical response pattern of different classes of C-nociceptors to pruritogens and algogens. J. Neurophysiol..

[13] Gupta K., Harvima I.T. (2018). Mast cell-neural interactions contribute to pain and itch. Immunol. Rev..

[14] Meixiong J., Basso L., Dong X., Gaudenzio N. (2020). Nociceptor-Mast Cell Sensory Clusters as Regulators of Skin Homeostasis. Trends Neurosci..

[15] Veiga-Fernandes H., Mucida D. (2016). Neuro-Immune Interactions at Barrier Surfaces. Cell.

[16] Kabashima K., Honda T., Ginhoux F., Egawa G. (2019). The immunological anatomy of the skin. Nat. Rev. Immunol..

[17] Kolter J., Feuerstein R., Zeis P., Hagemeyer N., Paterson N., d’Errico P., Baasch S., Amann L., Masuda T., Losslein A. (2019). A Subset of Skin Macrophages Contributes to the Surveillance and Regeneration of Local Nerves. Immunity.

[18] Kobayashi T., Ricardo-Gonzalez R.R., Moro K. (2020). Skin-Resident Innate Lymphoid Cells—Cutaneous Innate Guardians and Regulators. Trends Immunol..

[19] Marshall A.S., Silva J.R., Bannerman C.A., Gilron I., Ghasemlou N. (2019). Skin-Resident gammadelta T Cells Exhibit Site-Specific Morphology and Activation States. J. Immunol. Res..

[20] Veiga-Fernandes H., Artis D. (2018). Neuronal-immune system cross-talk in homeostasis. Science.

[21] Blake K.J., Jiang X.R., Chiu I.M. (2019). Neuronal Regulation of Immunity in the Skin and Lungs. Trends Neurosci..

[22] Ordovas-Montanes J., Rakoff-Nahoum S., Huang S., Riol-Blanco L., Barreiro O., von Andrian U.H. (2015). The Regulation of Immunological Processes by Peripheral Neurons in Homeostasis and Disease. Trends Immunol..

[23] Choi J.E., Di Nardo A. (2018). Skin neurogenic inflammation. Seminars in Immunopathology.

[24] Vidal Yucha S.E., Tamamoto K.A., Kaplan D.L. (2019). The importance of the neuro-immuno-cutaneous system on human skin equivalent design. Cell Prolif..

[25] Dantzer R. (2018). Neuroimmune interactions: From the brain to the immune system and vice versa. Physiol. Rev..

[26] Botchkarev V.A., Yaar M., Peters E.M., Raychaudhuri S.P., Botchkareva N.V., Marconi A., Raychaudhuri S.K., Paus R., Pincelli C. (2006). Neurotrophins in skin biology and pathology. J. Investig. Dermatol..

[27] Russo A.F. (2017). Overview of Neuropeptides: Awakening the Senses?. Headache.

[28] Li W.W., Guo T.Z., Liang D.Y., Sun Y., Kingery W.S., Clark J.D. (2012). Substance P signaling controls mast cell activation, degranulation, and nociceptive sensitization in a rat fracture model of complex regional pain syndrome. Anesthesiology.

[29] Theoharides T.C. (2020). The impact of psychological stress on mast cells. Ann. Allergy Asthma Immunol..

[30] Schlereth T., Schukraft J., Kramer-Best H.H., Geber C., Ackermann T., Birklein F. (2016). Interaction of calcitonin gene related peptide (CGRP) and substance P (SP) in human skin. Neuropeptides.

[31] Bianchi M.E. (2007). DAMPs, PAMPs and alarmins: All we need to know about danger. J. Leukoc. Biol..

[32] Zindel J., Kubes P. (2020). DAMPs, PAMPs, and LAMPs in Immunity and Sterile Inflammation. Annu. Rev. Pathol..

[33] Gong T., Liu L., Jiang W., Zhou R. (2020). DAMP-sensing receptors in sterile inflammation and inflammatory diseases. Nat. Rev. Immunol..

[34] Verstrepen L., Bekaert T., Chau T.L., Tavernier J., Chariot A., Beyaert R. (2008). TLR-4, IL-1R and TNF-R signaling to NF-kappaB: Variations on a common theme. Cell Mol. Life Sci..

[35] Yu H., Lin L., Zhang Z., Zhang H., Hu H. (2020). Targeting NF-kappaB pathway for the therapy of diseases: Mechanism and clinical study. Signal Transduct. Target. Ther..

[36] Maier E., Werner D., Duschl A., Bohle B., Horejs-Hoeck J. (2014). Human Th2 but not Th9 cells release IL-31 in a STAT6/NF-kappaB-dependent way. J. Immunol..

[37] Roosterman D., Goerge T., Schneider S.W., Bunnett N.W., Steinhoff M. (2006). Neuronal control of skin function: The skin as a neuroimmunoendocrine organ. Physiol. Rev..

[38] Minnone G., De Benedetti F., Bracci-Laudiero L. (2017). NGF and Its Receptors in the Regulation of Inflammatory Response. Int. J. Mol. Sci..

[39] Marz P., Heese K., Dimitriades-Schmutz B., Rose-John S., Otten U. (1999). Role of interleukin-6 and soluble IL-6 receptor in region-specific induction of astrocytic differentiation and neurotrophin expression. Glia.

[40] Furue M., Yamamura K., Kido-Nakahara M., Nakahara T., Fukui Y. (2018). Emerging role of interleukin-31 and interleukin-31 receptor in pruritus in atopic dermatitis. Allergy.

[41] Stander S., Steinhoff M. (2002). Pathophysiology of pruritus in atopic dermatitis: An overview. Exp. Dermatol..

[42] Mochizuki H., Tashiro M., Kano M., Sakurada Y., Itoh M., Yanai K. (2003). Imaging of central itch modulation in the human brain using positron emission tomography. Pain.

[43] Najafi P., Dufor O., Ben Salem D., Misery L., Carre J.L. (2021). Itch processing in the brain. J. Eur. Acad. Dermatol. Venereol..

[44] Golpanian R.S., Kim H.S., Yosipovitch G. (2020). Effects of Stress on Itch. Clin. Ther..

[45] Kim H.J., Park J.B., Lee J.H., Kim I.H. (2016). How stress triggers itch: A preliminary study of the mechanism of stress-induced pruritus using fMRI. Int. J. Dermatol..

[46] Sevilla L.M., Perez P. (2018). Roles of the Glucocorticoid and Mineralocorticoid Receptors in Skin Pathophysiology. Int. J. Mol. Sci..

[47] Mancino G., Miro C., Di Cicco E., Dentice M. (2021). Thyroid hormone action in epidermal development and homeostasis and its implications in the pathophysiology of the skin. J. Endocrinol. Investig..

[48] Zouboulis C.C., Picardo M., Ju Q., Kurokawa I., Torocsik D., Biro T., Schneider M.R. (2016). Beyond acne: Current aspects of sebaceous gland biology and function. Rev. Endocr. Metab. Disord..

[49] Wertheimer E., Trebicz M., Eldar T., Gartsbein M., Nofeh-Moses S., Tennenbaum T. (2000). Differential roles of insulin receptor and insulin-like growth factor-1 receptor in differentiation of murine skin keratinocytes. J. Investig. Dermatol..

[50] Daye M., Temiz S.A., Isik B., Durduran Y. (2021). Relationship between acanthosis nigricans, acrochordon and metabolic syndrome in patients with lichen planus. Int. J. Clin. Pract..

[51] Saylam Kurtipek G., Cihan F.G., Erayman Demirbas S., Ataseven A. (2015). The Frequency of Autoimmune Thyroid Disease in Alopecia Areata and Vitiligo Patients. Biomed. Res. Int..

[52] Kubba R., Suh D.H. (2021). Insulin Resistance Associated Acne. Acne: Current Concepts and Management.

[53] Terao M., Katayama I. (2016). Local cortisol/corticosterone activation in skin physiology and pathology. J. Dermatol. Sci..

[54] Phan T.S., Schink L., Mann J., Merk V.M., Zwicky P., Mundt S., Simon D., Kulms D., Abraham S., Legler D.F. (2021). Keratinocytes control skin immune homeostasis through de novo-synthesized glucocorticoids. Sci. Adv..

[55] Mancino G., Sibilio A., Luongo C., Di Cicco E., Miro C., Cicatiello A.G., Nappi A., Sagliocchi S., Ambrosio R., De Stefano M.A. (2020). The Thyroid Hormone Inactivator Enzyme, Type 3 Deiodinase, Is Essential for Coordination of Keratinocyte Growth and Differentiation. Thyroid.

[56] Ceruti J.M., Leiros G.J., Balana M.E. (2018). Androgens and androgen receptor action in skin and hair follicles. Mol. Cell Endocrinol..

[57] Spencer R.L., Deak T. (2017). A users guide to HPA axis research. Physiol. Behav..

[58] Slominski A., Wortsman J., Luger T., Paus R., Solomon S. (2000). Corticotropin releasing hormone and proopiomelanocortin involvement in the cutaneous response to stress. Physiol. Rev..

[59] Slominski A.T., Manna P.R., Tuckey R.C. (2014). Cutaneous glucocorticosteroidogenesis: Securing local homeostasis and the skin integrity. Exp. Dermatol..

[60] Hannen R.F., Michael A.E., Jaulim A., Bhogal R., Burrin J.M., Philpott M.P. (2011). Steroid synthesis by primary human keratinocytes; implications for skin disease. Biochem. Biophys. Res. Commun..

[61] Bikle D.D. (2012). Vitamin D and the skin: Physiology and pathophysiology. Rev. Endocr. Metab. Disord..

[62] Samuel S., Sitrin M.D. (2008). Vitamin D’s role in cell proliferation and differentiation. Nutr. Rev..

[63] Demay M.B. (2012). The hair cycle and Vitamin D receptor. Arch. Biochem. Biophys..

[64] Schauber J., Dorschner R.A., Coda A.B., Büchau A.S., Liu P.T., Kiken D., Helfrich Y.R., Kang S., Elalieh H.Z., Steinmeyer A. (2007). Injury enhances TLR2 function and antimicrobial peptide expression through a vitamin D–dependent mechanism. J. Clin. Investig..

[65] Krishnan A.V., Feldman D. (2011). Mechanisms of the anti-cancer and anti-inflammatory actions of vitamin D. Annu. Rev. Pharmacol. Toxicol..

[66] Barrea L., Savanelli M.C., Di Somma C., Napolitano M., Megna M., Colao A., Savastano S. (2017). Vitamin D and its role in psoriasis: An overview of the dermatologist and nutritionist. Rev. Endocr. Metab. Disord..

[67] Kader S., Temiz S.A., Akdag T., Unlu A. (2021). Evaluation of vitamin D and calcium mineral metabolism in patients with chronic urticaria. Int. J. Med. Biochem..

[68] Ekiz Ö., Balta I., Şen B.B., Dikilitaş M.C., Özuğuz P., Rifaioğlu E.N. (2014). Vitamin D status in patients with rosacea. Cutan. Ocul. Toxicol..

[69] Bosko C.A. (2019). Skin Barrier Insights: From Bricks and Mortar to Molecules and Microbes. J. Drugs Dermatol..

[70] Juráňová J., Franková J., Ulrichová J. (2017). The role of keratinocytes in inflammation. J. Appl. Biomed..

[71] Abdallah F., Mijouin L., Pichon C. (2017). Skin Immune Landscape: Inside and Outside the Organism. Mediat. Inflamm..

[72] Nestle F.O., Di Meglio P., Qin J.Z., Nickoloff B.J. (2009). Skin immune sentinels in health and disease. Nat. Rev. Immunol..

[73] Girardi M., Lewis J.M., Filler R.B., Hayday A.C., Tigelaar R.E. (2006). Environmentally responsive and reversible regulation of epidermal barrier function by gammadelta T cells. J. Investig. Dermatol..

[74] Zmora N., Bashiardes S., Levy M., Elinav E. (2017). The Role of the Immune System in Metabolic Health and Disease. Cell Metab..

[75] McKenzie D.R., Hart R., Bah N., Ushakov D.S., Munoz-Ruiz M., Feederle R., Hayday A.C. (2022). Normality sensing licenses local T cells for innate-like tissue surveillance. Nat. Immunol..

[76] Quaresma J.A.S. (2019). Organization of the Skin Immune System and Compartmentalized Immune Responses in Infectious Diseases. Clin. Microbiol. Rev..

[77] Ono S., Kabashima K. (2015). Proposal of inducible skin-associated lymphoid tissue (iSALT). Exp. Dermatol..

[78] Gröne A. (2002). Keratinocytes and cytokines. Vet. Immunol. Immunopathol..

[79] Stoitzner P., Stössel H., Romani N., Pfaller K. (2002). A close-up view of migrating Langerhans cells in the skin. J. Investig. Dermatol..

[80] Suto H., Nakae S., Kakurai M., Sedgwick J.D., Tsai M., Galli S.J. (2006). Mast cell-associated TNF promotes dendritic cell migration. J. Immunol..

[81] Byrne S.N., Limón-Flores A.Y., Ullrich S.E. (2008). Mast cell migration from the skin to the draining lymph nodes upon ultraviolet irradiation represents a key step in the induction of immune suppression. J. Immunol..

[82] Masopust D., Schenkel J.M. (2013). The integration of T cell migration, differentiation and function. Nat. Rev. Immunol..

[83] Xu Q., Zheng J. (2021). γδT cells as a potential candidate contributing to the development of psoriatic cardiovascular disease. Clin. Exp. Dermatol..

[84] Elnabawi Y.A., Garshick M.S., Tawil M., Barrett T.J., Fisher E.A., Lo Sicco K., Neimann A.L., Scher J.U., Krueger J., Berger J.S. (2021). CCL20 in psoriasis: A potential biomarker of disease severity, inflammation, and impaired vascular health. J. Am. Acad. Dermatol..

[85] Furue M., Kadono T. (2017). Inflammatory skin march” in atopic dermatitis and psoriasis. Inflamm. Res..

[86] Hill D.A., Spergel J.M. (2018). The atopic march: Critical evidence and clinical relevance. Ann. Allergy Asthma Immunol..

[87] Worbs T., Hammerschmidt S.I., Forster R. (2017). Dendritic cell migration in health and disease. Nat. Rev. Immunol..

[88] Gregor C.E., Foeng J., Comerford I., McColl S.R. (2017). Chemokine-Driven CD4(+) T Cell Homing: New Concepts and Recent Advances. Adv. Immunol..

[89] Islam S.A., Luster A.D. (2012). T cell homing to epithelial barriers in allergic disease. Nat. Med..

[90] Campbell J.J., Ebsworth K., Ertl L.S., McMahon J.P., Newland D., Wang Y., Liu S., Miao Z., Dang T., Zhang P. (2017). IL-17-Secreting gammadelta T Cells Are Completely Dependent upon CCR6 for Homing to Inflamed Skin. J. Immunol..

[91] Czarnowicki T., He H.Y., Wen H.C., Hashim P.W., Nia J.K., Malik K., Estrada Y., Kimmel G.W., Taliercio M., Krueger J.G. (2018). Alopecia areata is characterized by expansion of circulating Th2/Tc2/Th22, within the skin-homing and systemic T-cell populations. Allergy.

[92] Li S., Zhu G., Yang Y., Jian Z., Guo S., Dai W., Shi Q., Ge R., Ma J., Liu L. (2017). Oxidative stress drives CD8(+) T-cell skin trafficking in patients with vitiligo through CXCL16 upregulation by activating the unfolded protein response in keratinocytes. J. Allergy Clin. Immunol..

[93] Gazi U., Gureser A.S., Oztekin A., Karasartova D., Kosar-Acar N., Derici M.K., Artuz F., Mumcuoglu K.Y., Taylan-Ozkan A. (2019). Skin-homing T-cell responses associated with Demodex infestation and rosacea. Parasite Immunol..

[94] Yumeen S., Girardi M. (2020). Insights Into the Molecular and Cellular Underpinnings of Cutaneous T Cell Lymphoma. Yale J. Biol. Med..

[95] Saraceno R., Kleyn C., Terenghi G., Griffiths C. (2006). The role of neuropeptides in psoriasis. Br. J. Dermatol..

[96] Martins A.M., Ascenso A., Ribeiro H.M., Marto J. (2020). The Brain–skin connection and the pathogenesis of psoriasis: A review with a focus on the serotonergic system. Cells.

[97] Rousset L., Halioua B. (2018). Stress and psoriasis. Int. J. Dermatol..

[98] Schwab V.D., Sulk M., Seeliger S., Nowak P., Aubert J., Mess C., Rivier M., Carlavan I., Rossio P., Metze D. (2011). Neurovascular and neuroimmune aspects in the pathophysiology of rosacea. J. Investig. Dermatol. Symp. Proc..

[99] Muto Y., Wang Z., Vanderberghe M., Two A., Gallo R.L., Di Nardo A. (2014). Mast cells are key mediators of cathelicidin-initiated skin inflammation in rosacea. J. Investig. Dermatol..

[100] Rainer B.M., Kang S., Chien A.L. (2017). Rosacea: Epidemiology, pathogenesis, and treatment. Dermatoendocrinol.

[101] Kulka M., Sheen C.H., Tancowny B.P., Grammer L.C., Schleimer R.P. (2008). Neuropeptides activate human mast cell degranulation and chemokine production. Immunology.

[102] Meylan E., Tschopp J., Karin M. (2006). Intracellular pattern recognition receptors in the host response. Nature.

[103] Yoon S.H., Hwang I., Lee E., Cho H.J., Ryu J.H., Kim T.G., Yu J.W. (2021). Antimicrobial Peptide LL-37 Drives Rosacea-Like Skin Inflammation in an NLRP3-Dependent Manner. J. Investig. Dermatol..

[104] Yamasaki K., Di Nardo A., Bardan A., Murakami M., Ohtake T., Coda A., Dorschner R.A., Bonnart C., Descargues P., Hovnanian A. (2007). Increased serine protease activity and cathelicidin promotes skin inflammation in rosacea. Nat. Med..

[105] Kono M., Nagata H., Umemura S., Kawana S., Osamura R.Y. (2001). In situ expression of corticotropin-releasing hormone (CRH) and proopiomelanocortin (POMC) genes in human skin. FASEB J..

[106] Fimmel S., Glass E., Zouboulis C. (2005). Neuropeptides and UV radiation are possible mediators of inflammation in rosacea. J. Investig. Dermatol..

[107] Fimmel S., Abdel-Naser M.B., Kutzner H., Kligman A.M., Zouboulis C.C. (2008). New aspects of the pathogenesis of rosacea. Drug Discov. Today Dis. Mech..

[108] Wang W., Nan X., Ji P., Dow K. (2007). Corticotropin releasing hormone modulates endotoxin-induced inflammatory cytokine expression in human trophoblast cells. Placenta.

[109] Jamieson B.B., Kim J.S., Iremonger K.J. (2021). Cannabinoid and vanilloid pathways mediate opposing forms of synaptic plasticity in corticotropin-releasing hormone neurons. J. Neuroendocrinol..

[110] Suresh R., Mosser D.M. (2013). Pattern recognition receptors in innate immunity, host defense, and immunopathology. Adv. Physiol. Educ..

[111] Paus R., Theoharides T.C., Arck P.C. (2006). Neuroimmunoendocrine circuitry of the ‘brain-skin connection’. Trends Immunol..

